# Impact of Community Based Peer Support in Type 2 Diabetes: A Cluster Randomised Controlled Trial of Individual and/or Group Approaches

**DOI:** 10.1371/journal.pone.0120277

**Published:** 2015-03-18

**Authors:** David Simmons, A. Toby Prevost, Chris Bunn, Daniel Holman, Richard A. Parker, Simon Cohn, Sarah Donald, Charlotte A. M. Paddison, Candice Ward, Peter Robins, Jonathan Graffy

**Affiliations:** 1 Institute of Metabolic Science, Cambridge University Hospitals NHS Foundation Trust, Cambridge, Cambridgeshire, England; 2 Primary Care Unit, Department of Public Health and Primary Care, University of Cambridge, Cambridge, Cambridgeshire, England; Institute of Endocrinology and Metabolism, IRAN, ISLAMIC REPUBLIC OF

## Abstract

**Background:**

Diabetes peer support, where one person with diabetes helps guide and support others, has been proposed as a way to improve diabetes management. We have tested whether different diabetes peer support strategies can improve metabolic and/or psychological outcomes.

**Methods:**

People with type 2 diabetes (n = 1,299) were invited to participate as either ‘peer’ or ‘peer support facilitator’ (PSF) in a 2x2 factorial randomised cluster controlled trial across rural communities (130 clusters) in England. Peer support was delivered over 8–12 months by trained PSFs, supported by monthly meetings with a diabetes educator. Primary end point was HbA1c. Secondary outcomes included quality of life, diabetes distress, blood pressure, waist, total cholesterol and weight. Outcome assessors and investigators were masked to arm allocation. Main factors were 1:1 or group intervention. Analysis was by intention-to-treat adjusting for baseline.

**Results:**

The 4 arms were well matched (Group n = 330, 1:1(individual) n = 325, combined n = 322, control n = 322); 1035 (79•7%) completed the mid-point postal questionnaire and 1064 (81•9%) had a final HbA1c. A limitation was that although 92.6% PSFs and peers were in telephone contact, only 61.4% of intervention participants attended a face to face session. Mean baseline HbA1c was 57 mmol/mol (7•4%), with no significant change across arms. Follow up systolic blood pressure was 2•3mm Hg (0.6 to 4.0) lower among those allocated group peer-support and 3•0mm Hg (1.1 to 5.0) lower if the group support was attended at least once. There was no impact on other outcomes by intention to treat or significant differences between arms in self-reported adherence or medication.

**Conclusions:**

Group diabetes peer support over 8–12 months was associated with a small improvement in blood pressure but no other significant outcomes. Long term benefits should be investigated.

**Trial Registration:**

ISRCTN.com ISRCTN6696362166963621

## Introduction

Diabetes leads to morbidity and premature death [1] and is associated with depression, distress and reduced quality of life [2–4]. A range of personal, psychological, social and organizational barriers to diabetes care have been identified [5]. Behavioral interventions attempting to address metabolic, mental health and psychological issues have shown varying success [6].

One group of interventions to improve diabetes outcomes involves assistance from another person with the condition: such ‘peer supporters’ have often faced similar problems [7]. Support is characterised by empathy, and an approach that shares practical aspects of managing diabetes in day-to-day life i.e. the ‘how to do’ rather than the ‘what to do’. Peer support can involve individual or group approaches face-to-face, telephone and internet contacts [8].

Randomised controlled trial (RCT) evidence of a beneficial effect from peer support in diabetes remains inconsistent, with possible influences on biomedical outcomes such as glucose control, blood pressure (BP), dyslipidaemia and weight as well as mental health outcomes such as depression and diabetes distress [9]. However, these trials had limitations, differences in design and the extent of implementation extent [9]. Peers for Progress recently funded RCTs of peer support across 8 sites [10]. The largest of these, the randomised controlled trial of Peer Support In type 2 Diabetes (RAPSID), was designed to test the efficacy of individual and/or group peer support, within the context of a health system providing comprehensive primary care.

The study interventions were successfully piloted and demonstrated that the overall study design was appropriate for roll out into an RCT [11]. However, during the pilot, there was a tendency for the peers selected to be trained to adopt a ‘quasi health professional’ role (‘put on a pedestal’), rather than the desired non directive, facilitatory role. To address this, it was decided to firstly change the title from “peer supporter” to “peer support facilitator” (PSF) and secondly to replace the GP/practice nurse recommendation approach to asking all participants for an expression of interest to be a PSF. The second change, to address delays in the delivery of the PSF training by requests for diabetes education, was to provide diabetes education prior to the training of PSFs.

Both these recommendations required some operational changes and were fully implemented. We now describe the results of the RCT comparing different diabetes peer support strategies.

## Methods

The protocol for this trial and supporting CONSORT 2010 checklist are available as supporting information; see S1 Protocol and S1 CONSORT Checklist.

### Design Overview, setting and participants

RAPSID (ISRCTN66963621) was a 2x2 factorial cluster RCT comparing 4 intervention groups: Controls, 1:1 (individual) peer support group peer support, or combined group and 1:1 peer support among patients with type 2 diabetes. Participants had their diabetes for at least 12 months and those with dementia or psychotic illness were excluded. Participants were recruited from communities across Cambridgeshire and neighbouring areas of Essex and Hertfordshire. Communities were defined by local government (‘parish council’) boundaries. Registration was delayed until after recruitment had commenced for internal administrative reasons.

Recruitment commenced with posters at community venues, followed by mail invitations through 62 general practices, a hospital clinic and Diabetes UK members. Potential participants were mailed three sequential invitations to express an interest in joining the trial and finding out about becoming a peer support facilitator (PSF) i.e. a person who supported others. Attendance at a local venue was arranged. The patient recruitment phase was from 02/06/11 to 12/04/12, and follow-up from 10/09/12 to 29/08/13.

### Randomization and Interventions

All participants were invited to a 3·5 hour group education workshop, facilitated by a diabetes educator. This was to allow participants to begin the study with a reasonable understanding of diabetes, and focus the trial their participation in the trial on peer support rather than diabetes education. Attendance at the workshop was not compulsory for continuation in the trial.

Clusters were then randomised electronically in blocks of four (one cluster in each arm) by the statistician who had no trial involvement. Randomisation occurred once all clusters in the block were ready to proceed. All measurement staff were blind to the randomisation.

Table 1 describes PSF selection, the 2-day training and PSF support arrangements. The approach of selecting PSFs from those expressing an interest, rather than by nomination, was developed after the pilot study found that inviting general practitioners to nominate PSFs made them seem less like peers and more like health workers [11]. PSFs were normally from the same local community as their peers, in a ratio of approximately 1:5, with clusters including up to 15 participants in total. Where there were insufficient volunteers for the PSF role, further participants from within the cluster were approached, or those from other areas (in the same arm) were asked to assist.

**Table 1:** PSF selection, training and support programme and Education session content.

**PSF Selection**
PSFs were selected through:
Postal expression of interest
Not flagged as unsuitable by their general practice team
Unremarkable Criminal Record Bureau check
Attended the education session
Appeared flexible, adaptable and non-judgemental to observers at the measurement, education and training sessions
PSF training
Training of PSFs was undertaken separately for the 3 intervention programmes, lasted 2 days and included
An introduction to the trial
A session exploring the role of the peer supporter
Motivational interviewing techniques
Communication with health professionals
Confidentiality and data protection
A series of role-plays to practice boundary-setting, effective listening, dealing with difficult situations such as depression or alcoholism, and the limitations of the role (i.e. not offering knowledge or diagnosing problems)
Safety (e.g. lone worker policy if 1:1 or combined peer support arms)
Leading a group (if group or combined peer support arms)
Provision of a programme manual and a booklet describing local services
Duration of meetings (1:1 meetings-up to 1 hour; group meetings up to 1·5 hours.)
PSF support
Monthly meetings with a RAPSID Nurse
Bimonthly newsletters
Telephone access to a RAPSID nurse between meetings
Education Session content (all participants)
Identifying carbohydrates and understanding portions
Truths and myths about diabetes
Know your numbers and medications
Keeping active and looking after your feet)
Questions and answers

The intervention was delivered in 2 phases: an initial 4–6 months discussing 3 core aspects: (1) how to address barriers to care/practical issues arising from living with diabetes (2) social and emotional aspects of diabetes and (3) the health care received. PSFs were asked to be non-directive and deploy the listening skills explored during the PSF training in order to support peers in their efforts to attain better control over their diabetes and its effects on everyday life. In the second phase, PSFs were invited to continue with the same themes, but to discuss other topics not yet covered and consider inviting speakers.

PSFs were offered a mobile phone and reimbursed for hiring local venues for intervention sessions and refreshments. A ‘RAPSID nurse’ met with groups of PSFs within each intervention arm, in each of four geographical areas on a monthly basis. These meetings enabled PSFs to share positive and challenging experiences, generate potential solutions, discuss clinical issues that arose and keep the delivered content of the interventions in a standardised form. A RAPSID nurse was also contactable by telephone during office hours if PSFs had pressing concerns. Finally, an independent committee of four diabetes patients reviewed all study procedures and provided the study team with advice where needed.

PSFs were asked to keep records of telephone contacts and meetings with their peers. They were also provided with diaries and encouraged to write reflections on their experiences of delivering the intervention. Even if a peer was unable to attend a meeting, PSFs were asked to attempt to make contact and discuss arrangements. Contact between peers within the same trial arm was not recorded. Throughout the trial, care was taken not to introduce those in different arms of the study to each other. Costs were collected from PSF claims and invoices.

### Outcomes and Follow-up

A research nurse obtained consent, checked a self-completed questionnaire, measured weight, height, waist circumference, BP and collected blood (HbA1c, lipids) using standardised methodology/ equipment following training by the local Medical Research Council Epidemiology Unit. The questionnaire included socio-demographic and clinical questions (eg medications), and measures of depression (PHQ8), quality of life (EQ5D), diabetes self-efficacy, the Revised Diabetes Knowledge Scale (RDKS), diabetes distress, and medication adherence [12–17]. IFCC aligned HbA1c (High performance liquid chromatography, Tosoh G7, Tokyo, Japan) and lipid measurements (Dimension RxL Max Clinical Chemistry System, Siemens, Erlangen, Germany) were undertaken in one ‘CPA’ accredited laboratory to minimise variation in both the primary outcome, HbA1c and total cholesterol, a secondary outcome.

Outcomes were measured using postal questionnaires at 4–6 months and face-to-face measurements and questionnaires after 8–12 months. Clinical data was extracted where data remained missing, if collected within three months of the expected measurement date.

### Statistical Analysis

#### Sample size calculation

The trial tested the main effects of 1:1 peer support versus no 1:1 peer support (factor 1) and group versus no group peer support (factor 2). With a predicted mean cluster size of 10·6 participants and an intra-cluster correlation of 0·037 based upon an unpublished estimate from a previous study [18] for HbA1c, a design effect of 1·36 was anticipated. A sample size of 1,250 participants from 106 clusters, after allowing 6 clusters to drop out and a further 10% participant loss to follow-up, would leave 1,060 participants in 100 clusters for primary outcome analysis. The practical aspects relating to the number of likely available clusters, and numbers likely to be recruited from within a cluster, resulted in non-rounded power estimates. Based on a standard deviation for HbA1c of 1.25, this provided (two-sided tests, p<0·05), 91% power to detect a difference of 0·3% (3 mmol/mol) in mean HbA1c for each factorial main effect, 88% power to detect a difference of 0·4% (4 mmol/mol) between any two arms in the case of an unexpected interaction between the factorial effects [19], and 82% power to detect a 0·3% (3 mmol/mol) difference between combined intervention arms and the control arm. For questionnaire outcomes with the same intra-cluster correlation, based on 880 participants assuming a reduced 75% follow-up rate, there was 90% power to detect effect size differences of 0·25sd for factorial main effects, and 0·35sd for pair-wise comparisons. Where baseline of an outcome was collected, there was improved precision of intervention effects due to the adjustment for baseline in the analysis.

#### Statistics

Analyses were on an intention to treatment (ITT) basis, two-sided and assessed at p<0·05. Each continuous outcome was analysed using linear mixed effects regression models (using the nlme package in R software [20]) with cluster as the random effect, and adjusting for the baseline of the outcome using the missing indicator method to include any participants for whom the baseline was missing [21]. Homoscedastic variances were not assumed; but instead residual variances were allowed to be different within each trial arm. Main effects were included to test for the two factors after confirming no significant interaction between them [22]. These were then replaced by a term to contrast the combined intervention arms with the control arm to estimate the effect of any peer intervention. Patients with missing outcome data were excluded. A sensitivity analysis including all patients was conducted by using multiple imputation (based on 50 imputed datasets), which did not change the conclusions of the primary outcome analysis. Any missing outcome values were assumed to be missing at random. In order to understand the results of the ITT analysis more fully, an a priori per protocol (PP) population was defined by excluding those attending no meetings in the intervention arms. Attenders were significantly older, more highly educated, with lower body mass index (BMI) and smoking prevalence. A pre-planned subgroup analysis consisted of the same analyses but among participants with an HbA1c above 8% (64mmol/mol)).

All outliers of HbA1c >4 sd from the mean were included in the primary analysis, but then excluded as part of a sensitivity analysis. An additional sensitivity analysis involved performing the ITT analyses based on the per protocol population. SPSS version 21 was used for calculating descriptive statistics (IBM Corp, Armonk, NY, USA). Ethics approval was received from the Cambridgeshire 2 Research Ethics Committee (08/10/10), and all participants gave signed informed consent. The authors confirm that all ongoing and related trials for this intervention are registered.

## Results

### Recruitment

**Fig 1:**
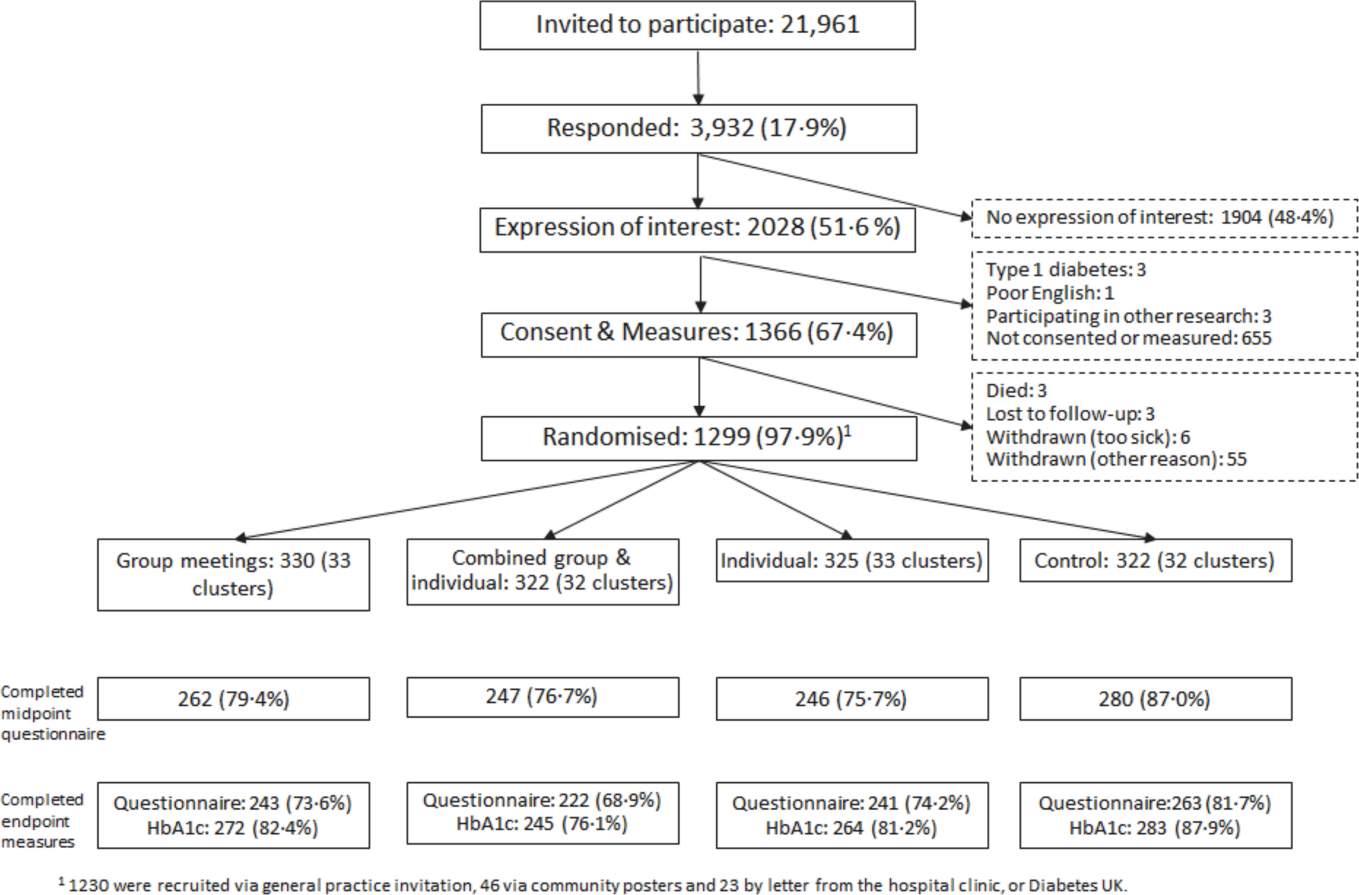
Recruitment and follow-up of participants.

The CONSORT diagram (Fig. 1) shows that recruitment and cluster number exceeded their targets. Follow-up met expectations. Baseline data for the 4 arms were well matched (Table 2). The majority reported antihypertensive (820; 63%), lipid-lowering (847; 65%) and diabetes medication (991; 76%). Compared with those without, those with an endpoint Hba1c were older (mean (sd) 65 (9) vs. 63(10) years p = 0·018), had longer diabetes duration (9 (11) vs. 8 (8) years p = 0.016), lower BMI (31.9 (6.8) vs. 33.6(6.7) kg/m2 p<0.001) and were more likely to be treated with anti-hyperglycaemic tablets (81.6% vs. 73.2% p = 0·004), hypertension treatment (66.2 vs. 59.4% p = 0·004) and dyslipidaemia treatment (68·5% vs. 60·7% p = 0·021) at baseline. The actual intra-cluster correlation coefficient at baseline was calculated to be 0.028 with a mean cluster size of 10 participants per cluster.

**Table 2:** Baseline characteristics of RAPSID participants allocated to individual, group, combined or control peer support intervention and intervention participation by cluster.

	Control (n = 322)	1:1 (n = 325)	Group (n = 330)	Combined (n = 322)
Males	191 (59·3%)	189 (58·2%)	216 (65·5%)	189 (58·7%)
Mean (SD) age (years)	64·6 (10·3)	65·2 (8·9)	65·2 (10·2)	65·3 (9·3)
Median (IQR) duration of diabetes (years)	6·5 (3·0–12·0)	7·0 (3·0–12·0)	7·0 (3·0–12·0)	6·0 (3·0–11·0)
Ethnic minority	22 (6·9%)	24 (7·5%)	23 (7·1%)	23 (7·3%)
Completed tertiary education	74 (24·6%)	82 (26·6%)	92 (29·8%)	77 (25·7%)
Professional/managerial	216 (68·6%)	204 (64·6%)	212 (67·1%)	209 (67·2%)
Married/cohabiting	239 (76·6%)	229 (72·0%)	250 (79·1%)	235 (76·8%)
Self-reported smoking	37 (11·8%)	28 (8·8%)	28 (8·8%)	26 (8·4%)
Insulin treated	47 (14·6%)	62 (19·1%)	53 (16·1%)	56 (17·4%)
Diabetes Tablets	252 (78·3%)	275 (84·6%)	260 (78·8%)	248 (77·0%)
Insulin and tablets	35 (10·9%)	53 (6·3%)	41 912·4%)	39 (12·1%)
Hypertension Treatment	192 (61·1%)	217 (68·0%)	202 (63·1%)	209 (67·4%)
Dyslipidaemia Treatment	198 (63·1%)	218 (68·3%)	213 (66·6%)	218 (70·3%)
BMI kg/m^2^	32·1 (6·1)	32·7 (6·4)	31·9 (5·8)	32·1 (5·8)
HbA1c mmol/mol	56·8 (12·7)	57·6 (13·3)	58·1 (13·0)	56·1 (12·8)
HbA1c %	7.3 (1.3)	7.4 (1.3)	7.5 (1.3)	7.3 (1.3)
SBP mm Hg	140·3 (18·1)/	140·6 (18·3)/	141·1 (17·3)/	139·4 (16·7)/
DBP mm Hg	75·5 (10·6)	76·1 (9·7)	75·6 (10·2)	76·4 (9·5)
Waist cm	108·5 (13·6)	110·0 (14·7)	109·6 (13·9)	108·6 (13·6)
Total Cholesterol mmol/l	4·41 (1·07)	4·39 (1·01)	4·33 (1·07)	4·41 (1·06)
Intervention/Participation				
Number of PSFs in cluster	0	61	57	49
Clusters with intervention	0/32	31/33	33/33	31/32
Contacted/Offered support	0	284(87·4%)	321(97·3%)	299(92·9%)
Attended at least once	0	175 (53·8%)	219 (66·4%)	198 (61·5%)
All 3 Peer support activities delivered#	0	31/33	30/33	29/32
Clusters with ≥5 months of intervention		26/33 (78.8%)	27/33 (81.8%)	25/32 (78.1%)

Numbers are n (%) or mean (SD);

# (1) how to address barriers to care/practical issues arising from living with diabetes (2) social and emotional aspects of diabetes and (3) the health care received

### Intervention

167 PSFs were trained, of whom, 127 facilitated at least one meeting. PSFs were available for 95/98 (96·9%) intervention clusters (Table 2). Table 1 also shows the number of clusters where interventions took place over ≥5 months. There was no significant difference between intervention arms in the number of months of intervention. Reporting data indicated that 90/98 (91·8%) intervention clusters covered the three core aspects of the intervention. Most (92·6%) peers and PSFs had been in mutual contact, but only 61·4% (592/977) of intervention participants attended an actual peer support session, with a mean number of attendances of 3·7, with others in telephone or e-mail contact. Reasons for non-attendance included the time to initiate local peer support (related to delays in honorary contract documentation, Criminal Record Bureau checks and training), sickness, changes in personal circumstances and moving house.

New activities initiated in the second phase of the intervention included table tennis, carpet bowling, golf and walking. Average estimated annual intervention costs per cluster are shown in Table 3. Costs varied widely, particularly venue costs.

**Table 3:** Costs of the intervention.

Item	Unit Cost (£)	Units	Total
***Peer support meeting costs***			
Venue (not 1:1)	£5·94	monthly	£71·28
Refreshments	£1·78	monthly	£21·36
Mobile top up vouchers	£3·23	monthly	£38·76
***Peer support facilitator meeting costs***			
RAPSID nurse time	£57·31	Monthly Up to 20 clusters	£34·39
RAPSID nurse travel costs	£3·01	Monthly Up to 20 clusters	£1·81
***Peer support facilitator training and material costs***			
PSF booklet and amenity booklet	£4·67	Per cluster	£4·67
PSF training trainer cost	£286·50	Per session Up to 10 clusters	£28·65
PSF training venue cost	£55·71	Per session Up to 10 clusters	£5·57
PSF training catering cost	£10·86	£10·86	£1·09
***Roll out costs***			***£207·58***

Assumes maximum number of clusters involved in training/PSF sessions With 15 participants attending/cluster = £13·84/participant/year Recruitment and coordination costs are not included Nurse is band 6 if works within a full multidisciplinary diabetes specialist service-otherwise band 7 Peer support meeting costs are from PSF claims Booklet costs include those given to PSFs who did not subsequently act as a PSF.

Few PSFs used the RAPSID mobile phones, preferring to use their own, so only the ongoing costs are included.

### Outcomes

There was greater follow up of HbA1c among the controls than the intervention arms (p = 0·002) (Fig. 1). Tables 4 and 5 show the ITT and per protocol analyses with no significant intervention effect on HbA1c, diastolic BP, weight, total cholesterol, diabetes knowledge, depression, quality of life, adherence or self-efficacy. However, the group main effect was associated with a significantly lower systolic BP (−2·31, 95% CI −4·01 to −0·61 mm Hg, p = 0·008). Among those attending at least once, the group main effect was associated with a −3x05 (95% CI −4·97 to −1·12, p = 0·002) mm Hg lower systolic and a −0·71 (95% CI −1·42 to −0·01, p = 0·048) cm lower waist circumference (although the waist results became −0·70 (95% CI −1·40 to 0·01) cm p = 0·053) after removing outliers more than 4 sd from the mean). Only 272 participants had an HbA1c above 8% (64mmol/mol) and no significant change was found in HbA1c (1:1 +0·59(95% CI −3·36 to 4·55); group +0·02 (95% CI −3·93 to 3·98); overall −0·02 (95% CI −4·57 to − 4·53) mmol/mol) or any of the other measures on follow up. There were no significant differences in self-reported medication changes between interventions. Diabetes distress improved significantly with the 1:1 main effect (−0·42 (95% CI −0·75 to −0·10, i.e. approximately 6% reduction) in the per protocol analysis.

**Table 4:** Anthropometric and Biochemical Outcomes at follow-up for RAPSID participants allocated to individual, group, combined or control peer support intervention.

					Intention to Treat—Per Protocol			(1 or more attendances)		
All	Control	1:1	Group	Combined	1:1 Effect	Group Effect	Any intervention	1:1 Effect	Group Effect	Any intervention
	N = 283* (N = 283)	N = 264 (N = 157)	N = 272 (N = 195)	N = 245 (N = 176)						
HbA1c follow up* §	59·7 (13·5)	60·3 (14·4)	60·0 (13·5)	58·8 (13·0)	0·19 (−1·13 to 1·51)	−0·17 (−1·49 to 1·14)	−0·29 (−1·77 to 1·20)	−0·50 (−1·91 to 0·91)	−0·77 (−2·17 to 0·63)	−0·98 (−2·41 to 0·45)
SBP follow up*	138·3 (16·8)	139·4 (16·2)	136·5 (16·3)	135·9 (16·5)	0·66 (−1·04 to 2·35)	−2·31 (−4·01 to −0·61)	−0·69 (−2·72 to 1·35)	0·96 (−1·00 to 2·92)	−3·05 (−4·97 to −1·12)	−1·17 (−3·31 to 0·98)
DBP follow up	75·2 (10·0)	74·8 (9·23)	73·9 (10·5)	75·0 (10·2)	0·05 (−1·04 to 1·13)	−0·46 (−1·55 to 0·62)	−0·66 (−1·89 to 0·58)	−0·31 (−1·55 to 0·94)	−1·14 (−2·38 to 0·11)	−1·09 (−2·43 to 0·26)
Pulse rate follow-up ¤	74·0 (13·9)	73·6 (12·9)	73·6 (12·8)	74·2 (12·2)	−0·46 (−1·80 to 0·88)	−0·19 (−1·52 to 1·15)	−0·75 (−2·31 to 0·81)	−0·02 (−0·11 to 0·06)	0·004 (−0·08 to 0·09)	0·03 (−0·06 to 0·12)
Total cholesterol follow-up*	4·21 (0·98)	4·25 (0·90)	4·17 (0·95)	4·15 (0·95)	−0·02 (−0·09 to 0·05)	−0·01 (−0·08 to 0·06)	0·01 (−0·07 to 0·09)	−0·60 (−2·17 to 0·97)	−0·22 −1·78 to 1·33)	−0·77 (−2·43 to 0·89)
Weight follow up*	90·0 (17·2)	92·1 (19·6)	90·9 (18·1)	89·2 (17·0)	0·08 (−0·53 to 0·70)	−0·09 (−0·70 to 0·53)	−0·22 (−0·97 to 0·54)	0·03 (−0·66 to 0·72)	−0·26 (−0·95 to 0·44)	−0·20 (−0·96 to 0·56)
Waist follow up* ¤	107·9 (13·6)	109·8 (14·8)	107·5 (13·1)	107·9 (13·4)	0·11 (−0·50 to 0·72)	−0·57 (−1·18 to 0·04)	−0·47 (−1·15 to 0·21)	0·43 (−0·26 1·13)	−0·71 (−1·42 to −0·01) **	−0·24 (−0·97 to 0·50)

*Sample size N refers to the primary outcome HbA1c in the Intention-to-treat population. The per protocol population is in brackets. Range of N for secondary outcomes are as follows

Control 240–291 (per protocol 240–291), 1:1 219–270 (141–159), Group 227–273 (174–195), Combined 202–257 (152–184)

** Result sensitive to outliers. Conclusion changed after removing outliers more than 4 sd from the mean. Group main effect became −0·70 (95% CI−1·40 to 0·01)

§ The model-based ICC for HbA1c at follow-up was calculated to be 0.043 for the primary Intention to treat analysis (including main effects terms only).

**Table 5:** Psychosocial Outcomes at baseline and change at follow-up for RAPSID participants allocated to individual, group, combined or control peer support intervention.

All	Control Baseline N = 322 (N = 322)	1:1 Baseline N = 325 (N = 175)	Group Baseline N = 330 (N = 219)	Combined Baseline N = 322 (N = 198)	1:1 Effect	Group Effect	Any intervention	Per protocol 1:1 Effect	Per protocol Group Effect	Per protocol Any intervention
Diabetes knowledge (0–15)	10·3 (3·03)	10·2 (2·96)	10·5 (3·00)	10·3 (3·20)	−0·13 (−0·47 to 0·21)	0·17 (−0·17 to 0·51)	0·05 (−0·35 to 0·45)	−0·05 (−0·44 to 0·33)	0·17 (−0·21 to 0·54)	0·20 (−0·19 to 0·59)
Depression PHQ8 (0–24)	4·49 (5·01)	4·39 (5·13)	4·49 (4·92)	4·59 (4·60)	−0·17 (−0·66 to 0·32)	−0·22 (−0·71 to 0·26)	−0·23 (−0·81 to 0·36)	−0·37(−0·96 to 0·22)	0·05 (−0·53 to 0·64)	−0·27(−0·90 to 0·36)
Diabetes Distress (4–24) DDS-4	6·61 (4·05)	6·53 (4·12)	6·27 (3·22)	6·71 (4·27)	−0·25 (−0·55 to 0·06)	0·13 (−0·18 to 0·43)	−0·11 (−0·46 to 0·25)	−0·43 (−0·76 to −0·10)	0·22 (−0·11 to 0·55)	−0·17(−0·53 to 0·200)
Quality of life EQ-5D (−0·11–1)	0·77 (0·27)	0·75 (0·30)	0·76 (0·26)	0·76 (0·27)	0·01 (−0·02 to 0·03)	0·00 (−0·02 to 0·02)	−0·01 (−0·03 to 0·02)	−0·00 (−0·03 to 0·03)	0·00 (−0·03 to 0·03)	−0·01 (−0·03 to 0·02)
Self-efficacy DSE-8 (8–80)	58·4 (17·2)	56·3 (18·2)	57·6 (16·2)	57·0 (17·1)	1·40 (−0·12 to 2·92)	−0·29 (−1·81 to 1·23)	1·62 (−0·11 to 3·35)	1·70 (−0·16 to 3·55)	−0·05 (−1·90 to 1·80)	1·80 (−0·17 to 3·77)
Morisky (Medical adherence) (0–4)	1·18 (1·12)	1·11 (1·14)	1·26 (1·14)	1·14 (1·12)	−0·02 (−0·14 to 0·10)	0·02 (−0·10 to 0·14)	−0·01 (−0·15 to 0·13)	−0·02(−0·15 to 0·12)	0·05 (−0·08 to 0·18)	−0·02(−0·16 to 0·13)

N refers to the maximum sample size in the Intention-to-treat population. Maximum sample size in the per protocol population is in brackets.

Range of N are as follows

Control 236–322 (per protocol 236–322), 1:1 215–325 (136–175), Group 212–330 (160–219), Combined 197–322 (145–198)

## Discussion

There was no significant change in HbA1c with any intervention. HbA1c was chosen as the primary outcome when the lower target for payments to general practitioners under the Quality and Outcomes Framework (QOF) was 7·0% (53mmol/mol) [23]. This QOF target was subsequently raised to 7·5% (58 mmol/mol): above the level for the majority of RAPSID participants (RAPSID mean HbA1c 57 mmol/mol (7·4%)) making achievement of a change unlikely. However, there was no change among those with an HbA1c ≥8% (≥64 mmol/mol) either. There were no significant differences between groups in change in self-reported diabetes medication, but detailed information on dose was not collected.

Group peer support was associated with a significant reduction in systolic BP, enhanced among those attending the intervention. This degree of reduction in BP (2–3mm Hg) would be associated with a relative reduction in myocardial infarction by 2–4%, stroke by 4–6% and all-cause mortality by 2–4% from observational data [24]. The mechanism for the BP reduction was unlikely to be increased medication adherence as the Morisky scale remained unchanged. It may be that the social support had direct effects on BP given the demonstrated links between the two [25]. Another possibility is that there may have been a change in lifestyle as suggested by the reduction in waist circumference observed in those who attended meetings. However, we found that those attending the intervention, and therefore belonging to the per protocol population, had significantly lower waist circumference at baseline (mean 108.3 versus 111.0, t-test p = 0.005) suggesting lifestyle change may have contributed to the observed difference, but no objective measures were used to assess this. Increased physical activity has previously been found to be associated with a 3–5 mm Hg drop in systolic BP, without a significant drop in diastolic BP [26]. There was no significant change in any of the other secondary outcomes.

Amongst those attending the 1:1 intervention (but not by intention to treat), there was a significant reduction in diabetes distress. Why this occurred in the 1:1 group is unclear and warrants further work. There was no significant change in any of the other secondary outcomes with the 1:1 peer support.

There are various reasons why the group approach may have been more effective than the 1:1 approach. These include the greater participation rates, the ability within a group for participants to select the members with whom they prefer to establish supportive relationships (whereas in a 1:1 interaction the relationship is dictated by study allocation), and also the observation that some groups undertook physical activity together.

### Comparison with other studies

This is the largest ever RCT of diabetes peer support and the only study investigating both 1:1 and group peer support approaches. It is one of the few truly community action, rather than health service/primary care, based RCTs. An impact on BP has been shown in some other studies [9]. One major Irish RCT in primary care demonstrated no significant metabolic impact [27]: although the BP dropped 3–4 mm Hg in their intervention group, it was not statistically significant. A comparison between nurse management and peer support also showed a non-significant blood pressure reduction with peer support [28]. The recent RCT of 1:1 telephone support in Hong Kong showed no benefits across the intervention cohort [29].

RAPSID included a cohort of patients who had metabolic results below or near national targets [23] had good access to primary care, were largely well-educated, and often from managerial/professional occupations. How the intervention would impact on more disadvantaged patients in the UK is unclear, but improvements in HbA1c have been shown among patients with poor glucose control and diminished access to health care elsewhere [30], even when compared with financial incentives [31].

### Strengths and limitations

RAPSID was well-powered to test its hypothesis, not only because it achieved its recruitment targets and size, but also because of high retention rates. The intervention was largely implemented having established peer support and delivered the planned content in almost all areas. However, a significant proportion of those invited did not participate and many participants did not take up the peer support, in spite of signing up to do so, and being contacted to arrange attendance. There were a range of hurdles to establishing peer support. In the 1:1 and combined interventions, there were 3 clusters in which no PSF could be identified, and in some clusters, there were insufficient to deliver the 1:1 intervention. Arranging a convenient time to meet was often a challenge, particularly for the group arms, addressed by other trials through telephone rather than face to face support [8]. A major hurdle was the time between the expression of interest and first intervention contact—this was largely due to the need to recruit sufficient numbers for the cluster including the need to identify, train and ‘check’ the PSFs. By the time the intervention was due to start, some participants had found other activities to interest them, while others were unwell or had moved. Under 10% of those originally invited expressed an interest in participating. We feel that this reflects that participation in RCTs and/or peer support are not for all of those with type 2 diabetes: further research is needed to assess reach outside of the RCT setting. However, in spite of the reduced uptake of the intervention within the trial, there was still a significant reduction in systolic blood pressure, and it is conceivable that greater uptake could have been associated with a greater impact. Either way, implemented in the real world, this trial did not deliver an effect on HbA1c or indeed the other secondary outcomes besides systolic blood pressure.

Even if participants attended the intervention, a significant proportion dropped out after each meeting. This did not necessarily mean that peer support halted, as there was undocumented evidence that contact between peers, and between peer and PSF, sometimes continued. The effect of peer support is likely to be dose dependent, but it was not possible to reliably assess the relationship between attendance and outcome within our study, because attendance was only observed post-randomisation and participants were obviously not randomised according to attendance level.

The probability of observing at least one false significant result was increased due to the relatively high number of secondary outcomes analysed. The duration of the trial was relatively short and impact over time may affect participants more or less, and in other ways, than found in RAPSID.

## Conclusion

Group, rather than 1:1 peer support, implemented with well-trained PSFs, supported and linked with health services through an experienced diabetes educator, reduced systolic BP, but not the primary outcome HbA1c, or other secondary outcomes, among English patients with relatively good metabolic control and access to health care. Further, well-powered, longer term efficacy and effectiveness studies would be helpful to confirm these findings and assess possible benefits over time.

## Supporting Information

S1 CONSORT Checklist CONSORT 2010 Checklist. (DOC)

S1 Protocol (PDF)
