# Supplementary material for: Impact of Community Based Peer Support in Type 2 Diabetes: A Cluster Randomised Controlled Trial of Individual and/or Group Approaches
Source: PLoS One. 2015 Mar 18;10(3):e0120277. doi: 10.1371/journal.pone.0120277 (PMC4364716; doi:10.1371/journal.pone.0120277)
Supplement: S1 Protocol — (PDF) [file pone.0120277.s002.pdf]

PROTOCOL

TITLE OF PROJECT

RAPSID: Can peer support, delivered as a group or individual intervention, enable people with diabetes and improve their health? (A RAndomised controlled trial of Peer Support In Type 2 Diabetes in Cambridgeshire, England)

OUTCOMES

Metabolic control
Quality of life
Self management behaviours
Self efficacy
Reach

NAME(S) OF PRINCIPAL INVESTIGATOR AND CO-INVESTIGATOR(S) (IF ANY)

PRINCIPAL INVESTIGATOR: DAVID SIMMONS
CO-PRINCIPAL INVESTIGATOR: JONATHAN GRAFFY
SIMON COHN
SARAH DONALD
PETER ROBINS
CHARLOTTE PADDISON
TOBY PREVOST
MARK EVANS
AMANDA ADLER
CATHY WALSH

BACKGROUND AND METHODS

A. BACKGROUND AND RATIONALE

Diabetes related damage is often now preventable with tight metabolic control (e.g. glucose, blood pressure, lipids), self care activities, regular review and timely intervention (1,2). However, preventable complications continue to occur (3), often due to personal and systems barriers to implementing diabetes care (4,5). Patients perceive psychological and psychosocial issues as important barriers to diabetes care, emphasizing in particular the strictness of the diabetes regimen, including diet, exercise and monitoring (6,7). In the DAWN study, self reported success with regimen adherence was relatively low in both Type 1 (46%) and Type 2 (39%) diabetes, but greater for self care than lifestyle behaviours (8). The need to overcome such barriers, so that people with diabetes are able to deal with the psychological, social and emotional issues that they face has become a major challenge.

A range of self management and structured educational programmes exist which emphasise empowerment and the pivotal role of the person with diabetes (9-12). This is a central theme within the Chronic Care Model for disease management (13) and the UK Diabetes National Service Framework (14). However, maintaining this role in managing an asymptomatic condition, on a day to day basis, with often unpleasant or obtrusive interventions, can be difficult. Different psychological interventions have been attempted to address this issue with varying success (15,16). Peer support, involving experience sharing, mentoring and role-modeling, has also been proposed as a way of overcoming some psychosocial barriers. Because peer supporters have faced many of the same problems, and the support offered relates to the task of managing diabetes in one's day to day life, peer support has the potential of being a practical way to address barriers which have been identified as so important in impeding successful diabetes self-management. A variety of individual and group approaches to providing peer support have been developed (9,17-19):

- ***Individual buddy approach:*** where a patient mentors and supports another patient.
- ***Individual expert patient approach:*** A structured group training programme providing "expert" patients with skills to train other patients in self management/problem solving.
- ***Traditional self help groups***: People meeting for mutual support in a relatively unstructured way. Such groups exist all over the world and although considered important, there has been little evaluation of whether they lead to behaviour change or improvements in health.
- ***Structured group education approach:*** Group education incorporating patients learning and supporting each other during the course (10,11).

Previous research suggests that peer support interventions are welcomed by participants, but has not provided robust evidence for its utility across all cultures (20). Two randomized trials have suggested improved self efficacy and HbA1c from peer-led self management support within Spanish-speaking communities (19) and increased physical activity from peer support amongst African-American women (21). Other trials are underway in Dublin (evaluating group support in primary care, including an educational component) (22) and Warwick (evaluating telecare by peers) (23). A model for how peer support could lead to sustainable behavioural changes that will improve long-term diabetes outcomes has been proposed by Heisler (17):

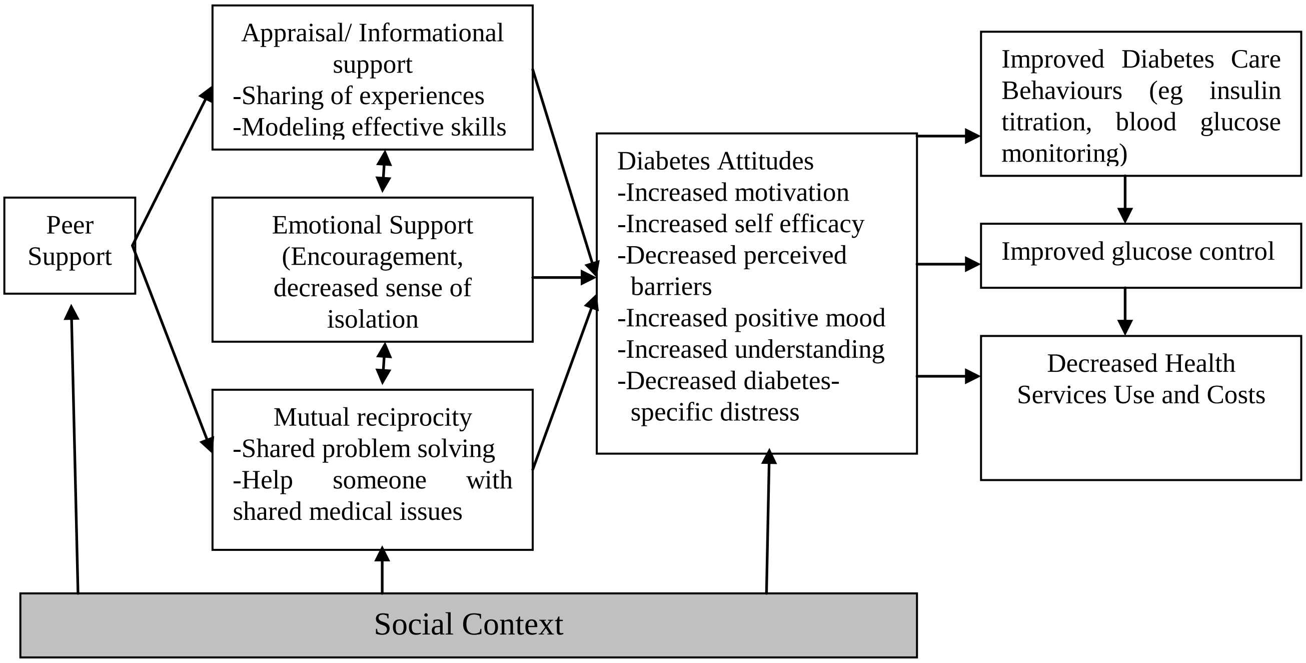

We wonder whether Heisler's model focuses on individual interactions between peer and person with diabetes, and understates the role of the context and social milieu in which the peer support occurs. This is a key component in Bandura's social cognitive theory (24), under which, people, environment and behaviour are all influencing each other. We have therefore included an additional construct of social context which is consistent with Bandura's Theory, can include 1:1 or group/community settings and which may be vital for some individuals. Bandura's approach could provide a framework (Appendix 1) to train peers for a support role, and also aid understanding of the ways in which peer support may be helpful (25). Aspects relevant to peer support include promoting self efficacy and motivation, buddying, mentoring and modeling within a social context which promotes and values skill acquisition, learning, skill implementation and coping.

We are unaware of studies which have compared individual and group approaches to peer support, and wonder whether patients vary in their responsiveness to such interventions. Groups could incorporate "social context", particularly if wider commonalities were present beyond diabetes (eg community links). We believe that comparing such approaches would assist in understanding the mechanisms behind any benefits or, alternatively, what the reasons are for any lack of benefit eg is it the patient mix, the intervention, the implementation of the intervention or even the conceptual framework. A concept analysis by Dennis has contributed to our thinking about the role of peer supporters and the training that they will need (26).

Heisler proposes that peer support can play an important role in identifying ways to overcome barriers to self care. We would agree with this and have created a framework (Appendix 2) to achieve this that has been used in New Zealand, Australia and the US (5,6,27,28). Also shown are examples of how the framework can be used to generate discussion between peer and person with diabetes in either a group or 1:1 situation.

We propose the following principles for the trial, beyond the pre-set requirements:

- Discussing and addressing barriers to self care should provide a context for appraisal and informational and emotional support.
- The trial should be able to combine evaluating the efficacy of the intervention with an evaluation of the mechanisms and issues behind the intervention. This would mean that even if the trial is negative, new knowledge would be gained for future trials.
- The trial should allow a comparison of the efficacy of 1:1 peer support, a group approach, a combined approach and normal care without peer support. We believe this question, of whether an individual or group approach is best is important and may remain unanswered unless a specific trial is undertaken
- The trial should assess uptake of group peer support and any wider population impact.

We have therefore devised a 2x2 factorial trial with an initial barriers survey and geographical clusters (defined by local government boundaries known as Parish Councils and including one or more villages or small towns) being allocated to neither intervention, a 1:1 peer support intervention, a community based peer support group intervention or both interventions.

B. PERTINENT PREVIOUS AND ONGOING WORK OF THE APPLICANT GROUP

David Simmons is the lead community diabetologist for Cambridgeshire, working between primary care and specialist services and therefore ideally placed for a community based trial of peer support. He has been involved in a range of community based research and service activities designed to increase peer and lay support for those with diabetes. This has included the establishment of the first South Asian diabetes support group in the United Kingdom in 1986 in Coventry which continues to this day. Attendance at the group was associated with improved knowledge and glycaemia (29). In New Zealand he established a lay diabetes community health worker (CHW) programme for the unemployed (30), introduced CHW interventions for the prevention of diabetes in high risk communities (largely Maori and Pacific family members (31,32), established 10 diabetes support groups for Maori, Pacific and rural

communities and helped evaluate a rural “diabetes club” where clinical care and peer support occurred concurrently (33). He has created a perceived “Barriers to Diabetes Care” evaluation framework used in New Zealand (5,6), rural Australia (27) and a deprived area of the US (28).

Jonathan Graffy has considerable experience in primary care research, on diabetes and more widely. This includes conducting one of the largest randomised trials of volunteer peer support (for breastfeeding mothers) (34). This has much in common with the planned diabetes trial and included a qualitative evaluation of participants’ perceived support needs (35). He is Clinical Lead for the East of England Primary Care Research Network (PCRN-EoE) which will engage general practices to recruit and follow up participants in the planned trial (www.pcrn-eoe.org). He is currently conducting research on patients’ experiences of diabetes care in local practices. He led the literature review group for the Dept of Health care planning policy report (36).

Sarah Donald and Mark Evans have expertise in group structured education programmes (DAFNE-Dose Adjustment For Normal Eating) with emphasis on self-management and support from group members. A component of the course focuses on goal setting and action planning, which patients discuss in pairs/groups independent of the trainer/healthcare professional. Our centre trains other healthcare professionals to deliver education programmes recognized by National Institute of Clinical Health and Excellence (NICE). We develop curriculae, training programmes and presentations used by other centres throughout the UK.

Simon Cohn, a medical anthropologist, has researched a wide range of topics relating to patients’ experience of living with chronic illness, their health behaviours, and their interactions with medical professionals. Previous publications include his initial PhD thesis investigating Type 2 diabetes patients’ understanding of the idea of empowerment (37), and relating to dietary adherence (38), risk perception(39); how patients with chronic illness understand medical knowledge, particularly issues of ambiguity and uncertainty (40, 41); how the diagnostic process can lead to a wide range of unintended individual and social consequences, for example in relation to psychiatric labels (42,43); how relationships between medics/

scientists and patients are frequently complex and multidirectional, involving negotiation and exchange (44,45). He has recently been appointed as Senior Lecturer within the General Practice and Primary Care Research Unit, as the first social scientist within the Dept Public Health Cambridge University.

Toby Prevost has been involved as a trial statistician in four complex intervention behavioural trials in Type 2 diabetes over the past eight years, and in cluster-randomised trials including the Cambridgeshire Addition trial (46). His statistical research has included investigating the suitability of methods for the design of cluster-randomised trials.

Charlotte Paddison is a research psychologist and has studied diabetes patients’ experiences with health services and the psychological impact of the diagnosis of diabetes on patients.

Peter Robins is a person with diabetes who has undertaken the DAFNE course. He is the Chair of the local Diabetes Care–Patient Advisory Committee (DCPAC) working to improve communication between patients, carers and health professionals and recommend improvements in diabetes care. He is a member of the Cambridgeshire Primary Care Trust Diabetes Education Working Group.

Amanda Adler helped develop type 2 diabetes risk models for use by health economists from the UKPDS (47). She is on the NICE Appraisals committee for new technologies and the chairperson of the NICE national committee to address effectiveness/cost-effectiveness of new type 2 diabetes agents. She has been involved in eg ADDITION (46), 4T (48) trials.

Cathy Walsh, Liaison Psychiatry consultant, is experienced in specific psychological interventions (eg motivational interviewing, CBT) and in assessing and treating people with diabetes who develop psychiatric problems (eg. anxiety, depression, adjustment disorders).

C. METHODS

Intended Audience and Setting

Our trial will focus on people with Type 2 diabetes across Cambridgeshire (population 587,573): Type 2 diabetes because it is more prevalent and because the experiences and needs

of people with Type 1 and Type 2 diabetes differ. Diabetes was recorded among 19,579 people aged 17+ years on local General Practice registers in March 2007 (49). The prevalence of diagnosed diabetes is 3.33%. The county includes over 80 towns and villages with Cambridge as the major town (population 120,000). The economic base comes from agricultural, science, educational and service industries with a large commuter population.

General care for diabetes available to Intended Audience

In the 2006 UK National Diabetes Survey, 89% of Cambridgeshire respondents reported that they received diabetes checkups at their general practice, 8% at hospital (50). The area includes two specialist diabetes services (one in Cambridge, one in Huntingdon), a community based diabetes support service including diabetes specialist nurses and dietitians and 76 general practices with primary care physicians and practice nurses providing structured diabetes care. All care is free at the point of care. The community diabetes services provide structured education for those with type 2 diabetes and in the 2006 Diabetes Survey 18% of all patients said they *"were offered or had participated in a course to help them manage their diabetes in the last 12 months"*. Insulin therapy is often commenced in primary care with or without the support of the community diabetes specialist nurses. General practices are computerized with comprehensive registers of patients with diabetes registered under a capitation system. All practices are funded to participate in the Quality Outcomes Framework (QOF) which includes reporting on a number of indicators relating to the quality of diabetes care across the practice (51). The service in Cambridge has a Diabetes Care Patient Advisory Committee (DCPAC), a group of volunteers with diabetes who have contributed to this proposal.

Recruitment or approaches to reaching intended audience

Two major recruitment methods into the trial will be used:

1 General practices possess lists of almost all local patients with diabetes and will be approached to send out an invitation to patients to participate in the trial. Practice registers will be screened for diabetes, excluding people known to have Type 1 diabetes, dementia, psychotic

illness or if it might be unsafe for a peer supporter to visit them at home. Invitation letters, and one reminder, will be sent to all residents with diabetes in smaller Parish Council areas, and a random sample of those with diabetes from larger areas anticipating that one third will join the trial, aiming for 20 people per cluster. Invitation letters will include the patient information sheet, consent form and a baseline survey relating to barriers to care (6). Participation rates will be maximized by using the Dillman method (52) in relation to format, inclusion of a stamped addressed envelope and follow up of the mail out. PCRN-EoE and Diabetes Research Network staff will support practice staff with recruitment.

2 <u>All of the villages and towns</u> have a means to contact local residents through notice boards, parish magazines, social clubs, Women's Institutes and local Parish Councils. These organizations will be approached to help invite people with diabetes into the trial. Materials distributed will include the same pack as supplied to patients invited by general practitioners. Participants recruited by community advertisement will also be screened.

Potential participants responding to the invitation will be contacted for an appointment for baseline assessment at a convenient venue (including the local general practice where appropriate). Participants will provide written informed consent at this assessment. The three towns with existing diabetes support groups will be excluded from the trial (approximately 25% of those with diabetes), but residents will be invited to contribute to training and back-up cover for the peer supporters recruited for the trial (Cambridge, Wisbech, Huntingdon).

We believe that there are a number of reasons why general practice teams, potential peer supporters and participants will be keen to participate in this trial.

- For general practices, this study has the potential to improve their patients' health by overcoming some of the barriers which frustrate current care. Local practices have responded enthusiastically to other diabetes studies (46, 53). Practical, governance and financial aspects

of study participation will be aided by PCRN-EoE, which will also be able to take over research nurse work on the study if needed.

- Potential peers will benefit from interesting and personally relevant training. Many people with diabetes are keen to share experiences and support others, but a particular advantage of doing so through this trial is that it will be provide a safe and supported framework for the peers. Their expenses will be paid, but they will not be paid for their time. Such a payment could undermine their role as “peers” who share common experiences with the other participants and could be viewed as against the “spirit of peer support” (22)).
- Potential participants will be individually invited by their general practitioner as well as through community networks. Peer support will be established on a local community basis and endorsed by local health services. Educational resources will be available to all participants and the trial will be run in a way to ensure that all participants know that their experiences and outcomes are important to the study team.

In view of the behavioural nature of the trial, and the need for a control group, mass media will not be approached, although if recruitment is low, this decision may need to be reviewed. In the trial documentation, participants will be advised that they will receive different forms of educational and/or personal support.

Approaches to implementing peer support programs that address the 3 core components:

We have devised a 2x2 factorial study design with geographical clusters allocated to neither intervention, a 1:1 peer support intervention, a community based peer support group intervention or both a 1:1 and group intervention. All three programmes would assist daily management and living with diabetes as well as social and emotional support by promoting self efficacy and providing buddying, mentoring and modeling. The group approach would also be able to provide wider social support. All participants will receive access to educational materials and normal care from their healthcare providers. The three intervention groups will draw their discussion topics from the barriers to diabetes care identified in the mail survey

(filtered to protect privacy in the group sessions), and also from a list of core topics for the programme to cover. Within the combined individual and group support arm of the trial, participants will be encouraged to agree which topics should be covered individually, and which should be discussed in the group sessions. The qualitative analysis will explore the impact of the different delivery modes on the content discussed and participants' perspectives on its value to them. We have summarised the way that the different intervention programmes will provide the three core components of peer support.

**Content:**	Educatn resource & normal care	Assist in daily manage-ment and living with diabetes	Social and emotional support	Social Contextual support	Linkage to clinical Care
**Interventn:**					
Individual 1:1 support	Yes	Sharing experiences & mentoring	Individual discussion of social and emotional aspects of living with diabetes	No	Individual review of care plan, linkage via Diabetes Specialist Nurse (DSN) if needed
Group support	Yes	Sharing experiences & co-mentoring in group	Group discussion of social and emotional aspects of living with diabetes	Yes	Group discussion of accessing services. Linkage via DSN if needed
Combined support	Yes	Sharing experiences & mentoring (group and/or individually)	Individual and/or group discussion of social and emotional aspects of living with diabetes	Yes	Both components As above.
Normal care	Yes	-	-		-

Delivering the individual 1:1 approach:

In the 1:1 peer approach, peers would be allocated a caseload of up to 10 individuals depending on their time. The peer would be from any of the 1:1 clusters, but resident as near to the participant as possible. Peers would follow their training in relation to the participant. Participants would be expected to have completed each aspect of the framework within 6 months.

Delivering the group approach:

The groups would be geographically based and therefore held largely within walking distance of the participant residence. Groups are expected to include 20 individuals on average, but this

will vary according to size of community. The size and dynamics of such groups will be allowed to evolve naturalistically, but will be described. Each group would have two leads, preferably from the cluster, although this may not be practicable for some of the smaller villages. A further volunteer would also be appointed and trained.   The group would progress through the barriers framework as with the 1:1 approach, but as a group rather than as individuals. Contact between meetings would generally be encouraged, to widen the peer support action.   The group would also be encouraged to develop wider social programmes within their village/town and to attract others with diabetes from the cluster who were not in the trial, providing it did not compromise the dynamics of the group. Alternatively, new groups could arise should membership become too large. Temporary group leaders and new leadership training may be needed in such instances.

Delivering the combined group and 1:1 approach

Peers would be trained to deliver both the 1:1 and group interventions. Participants who drop out of the groups would be followed up 1:1 by the trained peers. Individuals who require additional input beyond the groups would also be followed up in the 1:1 setting, comparable to the 1:1 approach..

The Peers

Patients consulted through the Diabetes Care Patient Advisory Committee (DCPAC) have made a number of recommendations regarding the peers:

- This is about patient support, not an educational intervention. Peers would need careful training in the peer support package about strict limits in terms of clinical management and “education”
- The peer would be somebody with diabetes, who is not a health professional, willing to help others understand their diabetes from the point of view of someone with diabetes.   The emphasis would be on how they have overcome their condition (i.e. they would need to be able to be a role model and have a pre-existing understanding of diabetes). It was felt that

such information given by a person with diabetes would be taken “in a different way”, would help encourage people to overcome difficulties and would come from a different perception of management than a health professional.

- The peer would need to be “like” the person being supported in relevant ways eg age, socioeconomic group. Geographically based groups would have this as a commonality. Young people would be likely to want a young peer/peer group
- Peers should be voluntary and could be expected to give 4-10 hours/week with no payment but costs covered. Peers would require “basic people skills” and would need careful selection.
- Support could be a few minutes on the telephone, face to face or any other way - this would be expected to evolve. Frequency of contact would depend on time available, need (i.e. baseline status) and how far down the support programme the participant was. Dependency would need to be avoided.

Peers will be sought from the recruited cohort as well as local diabetes services and primary care by both recommendation and invitation. Peers would need to come from intervention clusters (or outside the study area for 1:1 peers including Cambridge, Wisbech and Huntingdon). Criteria for selection will be developed. Participants who became peers would be excluded from the randomized trial (but part of a study of the impact on peers themselves). A succession strategy will ensure that if peer leaves there is more than one peer available. We will train reserve peers from outside the study area, or from areas with pre-existing support groups to provide back-up cover.

Peer Training

Training of peers would be undertaken separately for the 3 intervention programmes. This will cover: A) the theoretical basis of peer support and behaviour change interventions, drawing on Bandura’s framework (Appendix 1) the barriers framework (Appendix 2), and Heisler’s causal model for peer support (above); B) basic knowledge about diabetes including food, physical

activity, self-monitoring; C) group and/or individual support skills, including motivational interviewing techniques; D) approaches to ensure safety of the peer, (particularly for those in the 1:1 programmes), communication with health professionals, confidentiality and data protection and managing negative emotions and depression. The final curriculum would be developed in the first 8 months and include the core items agreed by the successful *Peers for Progress* applicants. Best practice examples of the curriculum components would be included from the applicant discussions and literature/available training manuals such as used for the Expert Patient Programme, the Goal Setting/Action Planning from DAFNE and the Irish RCT of peer support (22 and Appendix 3). We will use role play (e.g. "dealing with difficult customers"), video feedback and adult learning approaches as part of the training in group work and/or individual support skills. The training is anticipated to take 3-4 days with formative and summative assessments leading to the provision of training certificates. Monthly meetings with the Diabetes Specialist Nurse would take place for the first 6 months and every 2 months thereafter to provide further educational development and contribute to quality assurance.

Linkage with local clinical services

Peers will be linked with local services through:

- The training team will include members of the local diabetes and primary care services
- Supervision will be by a diabetes specialist nurse employed for this purpose and co-located with the local diabetes services. Supervision would include regular written reports and peer meetings. The DSN role may be shared to ensure their continuing local clinical practice, although this would need to be in a clinical role unlikely to contaminate those in the other limbs of the trial.
- Development of a referral pathway for patients reporting clinical difficulties. Generally, these would be discussed with the supervising DSN.

This linkage framework will be created in a way that should the trial show peer support to be successful and cost effective, the service that trains and supervises peers could be commissioned as part of the local diabetes and primary care services.

Intervention Timeline

The intervention will have 3 phases:

- Pilot stage to include formative evaluation of a short pilot in a non-study area (Cambridge city).
- First 6 months of the trial, implementing a structured programme on a monthly basis
- Second 6 months of the trial as a maintenance phase, where contact is maintained, but with a structure and frequency as agreed by the participants and peers.

The latter will link into a 6 month translation stage, to allow transition of the optimal trial approach to peer support to a wider community wide approach while some of the trial team remain. This is not funded within the current application and we will seek funding for this purpose from local sources.

Approaches to refining procedures and peer support intervention in months 1 – 8

Appendix 4 outlines the key activities in Months 1-8 including trial administration, intervention and evaluation. Of particular importance is the piloting of the peer intervention after the developmental work. The pilot study will be undertaken in months 6-8 in Cambridge, as it is not included in the study itself. Volunteers will be sought through local practices and the diabetes services to pilot the individual and group peer support interventions. After the 3 month trial period, participants may want the intervention to continue and this would provide an opportunity to develop strategies ahead of each stage of the trial.

Approaches to evaluation

Evaluation will include quantitative, qualitative and economic components collected through a range of methods to minimize measurement burden on participants and evaluation costs.

Quantitative analysis of trial outcomes:

Initial barriers mail survey during invitation into trial: This will be undertaken at the time of invitation into the trial and based upon prior studies (5,6). We expect to get some data on those who subsequently are not part of the trial. This will help us to assess the generalisability and reach of the intervention.

Baseline data collection: This will be at a local venue (e.g. general practice, community venue) and include consent to access participant health data (including metabolic and health utilization data). Key metabolic outcomes are HbA1c, lipids and blood pressure, weight and hypoglycaemia. HbA1c and lipids will be largely available from general practice/diabetes services as this is comprehensively (approximately 98%) collected through the Quality and Outcomes Framework for Diabetes. Where this is not collected, blood will be drawn. Blood pressure and weight will be measured in a standardized manner in view of the potential variability in quality from clinically colleted data. Information on hypoglycaemia will be collected by questionnaire. Health service utilization will be assessed through clinical records including ambulance use. Relevant demographic and health data will be collected. Specific questionnaires relating to self management will include those agreed across the Peers for Progress groups and likely to include questions relating to self-efficacy (54), diabetes self-care activities (55), medication adherence (56), family and friends subscale of the Chronic Illness Resources Survey (57). Quality of life will be assessed using the European Quality of Life-5 Dimensions (EQ 5D) which is well validated in the UK (58) and does not overburden participants.

6 months (mid point) data collection: This will include questionnaires completed by telephone and collection of existing health data

12 month (final) data collection: This will include comparable data to the baseline data collection.

Data collection: It is anticipated that some baseline and follow-up data will be collected by practice nurses at the participants' practices (working in separate measurement sessions), or by

research assistants employed by the Primary Care Research Network (Clinical Lead is J Graffy) or Diabetes Research Network (Primary Care Lead is S Griffin), both of which are keen to collaborate on this trial.

Qualitative analysis (Simon Cohn)

A key dimension of this proposal is the suggestion that the value of peer support, both one-to-one and in groups, is likely to be complex and multidimensional, and as a consequence difficult to assess solely using pre-determined and restrictive measures. Crucial to this study, therefore, will be the parallel collection of observation data from the peer training, debriefing and intervention sessions, the interview data from a sample of the participants, peers and project staff, in order to provide a more rich and nuanced record of both types of interventions. The aims of the qualitative analysis will be to explore the meaning of peer support as understood by peers and participants, their perspectives on the advantages and disadvantages of individual versus group support and ways that it might be improved. By analysing the data to establish common themes and concerns, we hope to be able to formulate a more sensitive understanding of the central mechanisms at play, and the significant differences when delivered to an individual compared with a group. The qualitative component will primarily be managed by S. Cohn, who has had extensive experience in similar research using qualified assistants. The data will be collected throughout the intervention period, providing a unique longitudinal record of the project as the interventions are delivered, and patients increasingly participate. Transcripts and notes will all be entered on NVivo data software to provide a common dataset that can be integrated with the quantitative and descriptive variables.

Economic analysis (Amanda Adler)

We will perform an economic evaluation to answer the following questions from the perspective of the National Health Service: Among individuals with diabetes in Cambridgeshire – (1) What are the costs associated with each method of peer support? (2) What is the change in effectiveness (HbA1c) and utility (quality of life) associated with each

method of peer support? (3) What are the costs associated with this change? We will measure utility using the EQ5D. We will model cost utility to estimate incremental cost effective ratios comparing 1:1 peer support to the absence of 1:1 peer support and group support to the absence of group support. We will use the UKPDS Outcomes model to assess potential years-of-life gained given changes in HbA1c.

Reach to and engagement of intended audience

The GP registers and Quality and Outcomes Framework data provide a denominator for the prevalence of diabetes across the county (3.3%). The local health board (the Primary Care Trust) has offered to assist with any data available. From this and local GP data (anonymised to avoid any privacy issues), we hope to be able to get a denominator for each cluster. This should allow an estimate of participation, both for the trial and uptake of peer support. Characteristics of those participating will be known from the baseline data collection. Some data will also be available on non-trial participants through the mail survey. Further data on individuals initially not engaging may become available in clusters randomly allocated to groups or both peer interventions through the local networking and de novo attendance at the groups. Such individuals will not be seen as part of the randomized controlled trial, but will be invited to be assessed through the trial and any changes assessed as a retrospective pre / post-intervention comparison. Peers will undergo the same measurements as the participants to assess any changes in a pre- post manner. This will allow assessment of the impact of the programme on their health and self-management behaviour.

With routinely collected clinical data (eg HbA1c, lipids, blood pressure) being so comprehensively collected under QoF, we would also hope to be able to assess any change in metabolic control for all of those with diabetes in the cluster. Again, we would find a way by which this data could be provided in an anonymised way. We would also be able to assess some changes in health service utilization within clusters (eg by assessing hospital admission rates by postcode). We will use the RE-AIM (reach, effectiveness, adoption, implementation,

and maintenance) framework which takes a broad perspective of issues that are important to policy makers to evaluate the public health benefits of the approaches tested (59).

Intervention fidelity

The extent, completeness and quality of the implementation of the intervention will be assessed during the above qualitative and quantitative evaluation, the peer-diabetes specialist nurse interactions, meeting records and activity logs prepared by the peer supporters. These will include attendance and the content of peer support discussions. We plan to develop this work in further discussions with Wendy Hardeman, a colleague in the Cambridge General Practice Research Unit (60).

Special issues

Reasons for drop out are particularly important and individuals will be carefully followed up and interviewed where possible. Family members and carers would be invited to participate into both 1:1 and group approaches through the person with diabetes. They would be asked to complete a questionnaire adapted from the patient questionnaire and would be included in the qualitative analyses.

Data management and analysis (Toby Prevost-statistician)

The trial design is randomised two-by-two factorial, testing main effects of Peer support versus no peer support (factor 1) and Group versus individual (factor 2). Randomisation to the four arms will be at the level of the village/area ("cluster"), with patients in the same village/area cluster randomised to arm. The justification for cluster randomisation is (1) to enable feasible group sizes in villages/area (2) to minimise contamination arising from contact between participants in the same village/area (3) bearing in mind that, within the two group arms and to a lesser extent in the peer-individual arm, clustering of outcomes would already exist even with an individual randomised design, reducing the marginal effect of cluster randomisation on sample size inflation. A restricted randomisation method that incorporates cluster-level stratifiers will be used, with randomisation performed centrally by a third party independent of

trial coordination, free from access to the study database, and with clusters identified by unique study numbers without access to village/area names. The primary outcome will be HbA1c. A plausible and achieved effect size difference in mean HbA1c in another study (19) is 0.36. We propose a realistic effect size in the range 0.3 to 0.4 in difference in mean HbA1c for each factor as worth detecting. A recent trial in patients recruited from Cambridgeshire and Oxfordshire (53) has demonstrated a standard deviation of HbA1c between patients of 1.25. The Cambridgeshire ADDITION trial provides a recent and local estimate of the intracluster correlation coefficient for HbA1c of 0.037 (46).

In the proposed study we anticipate being able to recruit a mean of 20 patients per randomised villages/area. Allowing for 10% dropout in access to records of HbA1c at study follow-up, we therefore anticipate a mean cluster size of 18 participants at the analysis stage. With 1520 participants recruited in 76 randomised village/area clusters (20 per cluster), and allowing for four clusters to withdraw from the study without allowing access to patient follow-up data, we anticipate that 1300 participants (90% of 1440) will be followed up with an HbA1c. There will be 90% power to detect a difference of 0.3 in mean HbA1c for each of the two factors at the 5% level of significance, allowing for a design effect due to clustering of 1.63, based on mean cluster size of 18 participants per cluster and ICC of 0.037. There will also be 90% power to detect a difference of 0.4 in mean HbA1c between any two of the four arms, and these comparisons offer protection against any unexpected interaction that might be observed between the two factors. As a sensitivity analysis for this sample size calculation, in the event of a higher observed ICC, for example arising from the interaction between grouped participants, this coefficient would need to be approximately twice as large (0.07) to be able to effect a reduction in power from the desired 90% to a minimally acceptable 80%. For context, the ICC in a previous trial was 0.047 (61)

	1:1 Peer Support	No 1:1 Peer support
Group	380 recruited<br>19 randomised areas<br>325 HbA1c follow-up<br>270 other outcomes follow-up<br>18 followed-up areas	380 recruited<br>19 randomised areas<br>325 HbA1c follow-up<br>270 other outcomes follow-up<br>18 followed-up areas
Not group	380 recruited<br>19 randomised areas<br>325 HbA1c follow-up<br>270 other outcomes follow-up<br>18 followed-up areas	380 recruited<br>19 randomised areas<br>325 HbA1c follow-up<br>270 other outcomes follow-up<br>18 followed-up areas

Other measures may not be able to be ascertained from medical records and a reduced follow-up rate of 75% from questionnaires is assumed. With 1080 followed-up (15 participants per cluster), there will be 90% power to detect sufficiently small effect size differences in each factor of 0.25sd for factorial main effects, and 0.35sd for pairwise comparisons between arms.

Statistical Analysis: All analyses will account for the clustered design through the use of linear mixed effects for modelling continuous outcomes and simple comparisons of proportions, and Generalised Estimating Equations with exchangeable correlation matrix for modeling binary outcomes. Where a baseline of an outcome is available there will be an adjustment for this covariate in order to improve the precision of the estimated intervention effects. Participants with a missing value for baseline will be retained using the method of White et al. The analysis will follow guidelines for the approach for factorial trials (62,63). The methods will be adapted to allow for varying intracluster correlation by arm where this is identified. The influence of missing data for the primary outcomes will be investigated using the multiple imputation method of Rubin outlined in Shafer and the primary intention to treat analysis will be supported by per protocol analysis (64,65). Further before-after analyses will allow assessment of change in those clusters involved in the wait-list design. All analyses will be two-sided and assessed at the 5% level of significance. An interim estimation of variances, cluster sizes, cluster withdrawal, and participant follow-up and questionnaire return rates will be made to ensure adequate study power to detect effect sizes in the stated range. A trial analysis plan will be developed prior to the analysis.

The following Sections D, E, and F do not count toward the 20-page limit for the previous sections.

D. READINESS TO PARTICIPATE IN COLLABORATIVE RESEARCH GROUP Experience with collaborative research or evaluation projects and readiness to collaborate with other investigators in (a) exchanging curricula and approaches to peer support, (b) identifying and implementing key common evaluation indicators (metabolic control, self management behaviors, quality of life), (c) collaborative publications reporting cross cutting themes of the research.

This is an experienced research group with wide experience in collaborative research and we are keen to collaborate with other groups.

A The group has been involved in curricula development in DAFNE (a national educational/empowerment programme in the UK), lay health worker training in New Zealand and peer support in breastfeeding and diabetes in the UK and New Zealand.

B The group has been involved in identifying key common evaluation indicators in metabolic control (eg in diabetes in pregnancy (66), in diabetes overall (67)), self management behaviours (eg readiness to change lifestyle (68), peer support for breastfeeding (35), Barriers to Diabetes Care tool in New Zealand, Australia and the United States (5,6,27,28), psychological factors (69)

.

C DS has worked with the University of Pittsburgh in an RCT based upon the Chronic Care Model using his barriers to care approach (70). JG is a member of the UK National School for Primary Care Research (NSPCR) Recruitment and Retention working group. We have recently submitted two papers on improving recruitment and retention to primary care studies (reviewing the literature and reporting a nominal group exercise with research staff). The *Peers for Progress* programme offers an ideal opportunity for a publication reviewing issues in recruitment to peer support studies.

E. IRB APPROVAL AND INFORMED CONSENT Provide a brief discussion about the ethical implications of this study, how human subjects will be protected, and regulations for assuring such protection that govern the applicants and their institutions.

1. The major ethical issue is around ensuring liaison with the usual health care providers and service purchasers with whom we already work.
2. A further issue relates to the end of the trial and how peer support might be integrated into normal care at the end of the trial if successful. (As peers are to remain unpaid, the only additional expenses would be operating costs, most of which would take place in the fist 6 months of the trial. The area has a Diabetes Care Patient Advisory Committee and discussions will be held depending on the outcome of the trial.)
3. Confidentiality by peer supporters. (Selection of peers should include a general practitioner reference or review of application. Peer training should cover confidentiality)
4. Confidentiality in support groups (Cover in peer training. Include in group rules at start of group)
5. Access to GP records. (Patients will be identified from searches of GP registers, conducted by practice staff, or by PCRN staff with honorary contracts form the practices to cover this. Invitation letters will be sent directly from practices, so recruitment data need not be

transferred out of the practices. Individual participants will then provide written consent for research staff to access their records for follow up).

6. We will ensure ongoing support for the peers, which will enable the team to identify and address unexpected ethical issues if they arise.

There is a fully functioning Ethics Committee in the area for protection of participants. We will apply for approval before commencing the research and meet all NHS ethics and Research Governance requirements.

F. REFERENCES Provide a bibliography of the references cited in the Background and Methods. The applicants may choose their preferred reference style but it should be clear and consistent in the application.

1. Williams R, Herman W, Kinmonth A-L, Wareham NJ (Eds). The Evidence Base for Diabetes Care. Wiley and Sons, Chichester, England, 2002.
2. IDF Clinical Guidelines Task Force. Global guideline for Type 2 diabetes. Brussels: International Diabetes Federation, 2005.
3. International Diabetes Federation. World Diabetes Atlas 2005. Brussels: International Diabetes Federation, 2005.
4. Hiss RG. Barriers to care in non-insulin-dependent diabetes mellitus: The Michigan experience. Ann Intern Med. 1996;124:146-148.
5. Simmons D, Weblemoe T, Voyle J, Prichard A, Leakehe L, Gatland B. Personal barriers to diabetes care: lessons from a multiethnic community in New Zealand. Diabet Med. 1998;15:958-964.
6. Simmons D, Lillis S, Swan J, Haar J. Discordance in perceptions of barriers to diabetes care between patients, primary care and secondary care. Diabetes Care 2007;30:490-5
7. Peyrot M, Rubin RR, Lauritzen T, Snoek FJ, Matthews DR, Skovlund SE, International DAWN Advisory Panel. Psychosocial problems and barriers to improved diabetes management: results of the Cross-National Diabetes Attitudes, Wishes and Needs (DAWN) Study. Diab Med 2005; 22:1379-1385
8. Peyrot M, Rubin RR, Lauritzen T, Snoek FJ, Matthews DR, Skovlund SE, International DAWN Advisory Panel. Psychosocial problems and barriers to improved diabetes

management: results of the Cross-National Diabetes Attitudes, Wishes and Needs (DAWN) Study. Diab Med 2005; 22:1379-1385

9. Funnell MM. Peer based behavioural strategies to improve chronic disease self management and clinical outcomes: evidence, logistics, evaluation considerations and needs for future research. Background paper 2 for the WHO Consultation on Peer Support programmes in diabetes, Geneva 5-7 November 2007.
10. National Institute for Clinical Excellence. The clinical effectiveness of diabetes education models for type 2 diabetes: a systematic review. *Health Technol Assess* 2008;**12**(9):1–136
11. National Institute for Clinical Excellence. TA60 Diabetes (types 1 and 2) – patient education models summary. http://www.nice.org.uk/guidance/index.jsp?action=byID&r=true&o=11496 (accessed 24/7/8)
12. Norris S, Engelgau M, Venkat Narayan K. Effectiveness of self management training in Type 2 diabetes. Diabetes Care 2001;24:561-587
13. Bodenheimer, T., Wagner, E. H., & Grumbach, K. 2002, "Improving primary care for patients with chronic illness", *JAMA*, vol. 288, no. 14, pp. 1775-1779.
14. Department of Health 2001, *National Service Framework for Diabetes: Standards*, Department of Health, London.
15. Ismail K, Winkley K, Rabe-Hesketh S. Systematic review and meta-analysis of randomised controlled trials of psychological interventions to improve glycaemic control in patients with Type 2 diabetes. Lancet. 2004; 363:1589-1597
16. Winkley K, Ismail K, Landau, Eisler I. Psychological interventions to improve glycaemic control in patients with type 1 diabetes: systematic review and meta-analysis of randomised controlled trials. Br med J 2006; 333:65-71.

17. Heisler M. Different models to mobilize peer support to improve chronic disease self management and clinical outcomes: evidence, logistics, evaluation considerations and needs for future research. Background paper 1 for the WHO Consultation on Peer Support programmes in diabetes, Geneva 5-7 November 2007.
18. Meakin J: With a little help from my peers. *Diabetes Update 2003(Autumn 2003)*18-21.
19. Lorig K, Ritter PL, Villa F, Piette JD. Spanish Diabetes Self-Management With and Without Automated Telephone Reinforcement. Diabetes Care 2008; 31:408-414
20. Lewin, S. A., Dick, J., Pond, P., Zwarenstein, M., Aja, G., van, W. B., Bosch-Capblanch, X., & Patrick, M. 2005, "Lay health workers in primary and community health care", *Cochrane.Database.Syst.Rev.* no. 1, p. CD004015.

21. Keyserling, T. C., Samuel-Hodge, C. D., Ammerman, A. S., Ainsworth, B. E., Henriquez-Roldan, C. F., Elasy, T. A., Skelly, A. H., Johnston, L. F., & Bangdiwala, S. I. 2002, "A Randomized Trial of an Intervention to Improve Self-Care Behaviors of African-American Women With Type 2 Diabetes: Impact on physical activity", *Diabetes Care*, vol. 25, no. 9, pp. 1576-1583.

22. Paul GM, Smith SM, Whitford DL, O'Shea E, O'Kelly F, O'Dowd T. Peer support in type 2 diabetes: a randomized controlled trial in primary care with parallel economic and qualitative analyses: pilot study and protocol. BMC Family Practice 2007;8: 45 doi:10.1186/1471-2296-8-45
23. Dale, J., Caramlau, I., Docherty, A., Sturt, J., & Hearnshaw, H. 2007, "Telecare motivational interviewing for diabetes patient education and support: a randomised controlled trial based in primary care comparing nurse and peer supporter delivery", *Trials*, vol. 8, no. 1, p. 18.

24. Bandura A. Self-efficacy mechanism in human agency. Am Psychol 1982;37:122-147.

25. Glanz, K., Rimer, B.K. & Lewis, F.M. (2002). *Health Behavior and Health Education. Theory, Research and Practice.* San Fransisco: Wiley & Sons.

26. Dennis, C. L. 2003, "Peer support within a health care context: a concept analysis", *International Journal of Nursing Studies*, vol. 40, no. 3, pp. 321-332.

27. Simmons D, Bourke L, Yau E, Hoodless M. Diabetes risk factors, diabetes and diabetes care in a rural Australian community. Aust J Rural Health 2007;15: 296-303

28. Piatt G, Anderson R, Simmons D Siminerio L, Zgibor J. Who Benefits Most from Diabetes Education? Results of a Randomized Controlled Trial. American Diabetes Association, Orlando, Florida, June 2004; 53(2): A90

29. Simmons D. Diabetes self help facilitated by local diabetes research: The Coventry Asian Diabetes Support Group. Diabet Med. 1992;9:866-869.

30. Tregonning PB, Simmons D, Fleming C. A community diabetes educator course for the unemployed in South Auckland, New Zealand. Diabetes Educ. 2001;27:94-100.

31. Simmons D, Fleming C, Voyle J, Fou F, Feo S, Gatland B. A pilot urban church-based programme to reduce risk factors for diabetes among Western Samoans in New Zealand. Diabet Med. 1998;15:136-142.

32. Simmons D, Rush E, Crook N. Development and piloting of a community health worker based intervention for the prevention of diabetes among New Zealand Maori in Te Wai o Rona: Diabetes Prevention Strategy. Public Health Nutrition (in press)

33. O'Grady A, Simmons D, Tupe S, Hewlett G. Effectiveness of changes in the delivery of diabetes care in a rural community. Aust J Rural Health. 2001;9:74-78.

34. Graffy, J., Taylor, J., Williams, A., & Eldridge, S. 2004, "Randomised controlled trial of support from volunteer counsellors for mothers considering breast feeding", *BMJ*, vol. 328, no. 7430, pp. 26-0.

35. Graffy, J. & Taylor, J. 2005, "What Information, Advice, and Support Do Women Want With Breastfeeding?", *BIRTH*, vol. 32, no. 3, pp. 179-186.

36. Department of Health and Diabetes UK Care Planning Working Group 2006, *Care Planning in Diabetes*, Department of Health, London.

37. The Discourse of Empowerment and Patient Self-Care, unpub. thesis, London University, 1995

38. Being Told What to Eat. In Food, Health and Identity ed. P.Caplan, Routledge 1997:193-213

39. Risk, ambiguity and the loss of moral control. In Risk Revisited, ed P. Caplan, Pluto Press, pp.1999: 204-226

40. 'Taking Time to Smell the Roses', Anthropology and Medicine, 1999 (6): 2.

41. Cohn, S., Dyson C., and Wessely S. 'Early Accounts of Gulf War Illness and the Construction of Narratives in UK Service Personnel.'. Plus commentary. Social Science and Medicine, proofs returned (2008)

42. 'Increasing Resolution, intensifying ambiguity', Economy and Society, 2004;33: 52 – 7

43. 'When Patients see their Mental Illness', In Technologized Images, Technologized Bodies: Anthropological Approaches to a New Politics of Vision, eds J Edwards, P Harvey and P Wade, Berghahn Press, in press

44. Cohn, S and Cullen., J. Making Sense of Mediated Information: Empowerment and Dependency. In Innovative Health Technologies, ed A. Webster. Palgrave 2006

45. Intimacy in the study of empathy, *History of the Human Sciences*, 2008;21:84-101

46. Eborall HC, Griffin SJ, Prevost AT, Kinmonth AL, French DP, Sutton S. Psychological impact of screening for type 2 diabetes: controlled trial and comparative study embedded in the ADDITION (Cambridge) randomized controlled trial. Br Med J 2007;335: 486-9

47. Stevens R, Kothari V, Adler AI, Stratton IM, Holman RR . The UKPDS risk engine: a model for the risk of coronary heart disease in type 2 diabetes. Clinical Science 101: 671-679, 2001

48. Holman RR, Thorne KI, **Farmer AJ**, Davies MJ, Keenan JF, Paul S, Levy JC, the 4T Study Group. Addition of Biphasic, Prandial, or Basal Insulin to Oral Therapy in Type 2 Diabetes. N Engl J Med 2007; 357: 1716-1730

49. Eastern Regional Public Health Observatory. *Trend in prevalence of COPD, CHD, Diabetes*, Hypertension, and Cancer at PCT level (QOF 2004/05 to 2006/07).* www.erpho.org.uk/viewResource.aspx?id=17532

50. Healthcare Commission. *Results of the national survey of people with diabetes*. www.healthcarecommission.org.uk/healthcareproviders/nationalfindings/surveys/healthcareproviders/surveysofpatients/longtermco/resultsofthe2006diabetessurvey.cfm

51. Gulliford MC, Ashworth M, Robotham D, Mohiddin A. Achievement of metabolic targets for diabetes by English primary care practices under a new system of incentives. Diabet Med 2007; 24(5):505-511.

52. Dillman D. Mail and internet surveys: the tailored design method. New York: Wiley; 2000

53. Farmer AJ, Prevost AT, Hardeman W, Craven A, Sutton S, Griffin SJ, Kinmonth AL: Support and Advice for Medication Trial Group. Protocol for SAMS (Support and Advice for Medication Study): a randomised controlled trial of an intervention to support patients with type 2 diabetes with adherence to medication. *BMC Family Practice* 2008;9:20.

54. McDowell J, Coutney M, Edwards H, Shortridge-Baggett L. Validation of the Australian/English Version of the Diabetes Management Self-Efficacy Scale. Int J Nurs Pract 2005; 11:177-194

55. Toobert D, Hampson S, Glasgow R: The summary of diabetes self care activities measure. Diabetes Care 2000;23:943-950

56. Horne R. Non-adherence to medication: causes and implications for care. In A Behavioural Approach to pharmacy practice. Edited by Gard P. Oxford, Blackwell 2001;111-130

57. Glasgow R, Toobert D, Barreera M, Strycker L. The Chronic Illness Resources Survey: Cross validation and sensitivity to intervention. Health Educ Res 2005;20:402-409

58. Glasziou P, Alexander J, Beller E, Clarke P, Group AC. Which health-related quality of life score? A comparison of alternative utility measures in patients with Type 2 diabetes in the ADVANCE trial. *Health & Quality of Life Outcomes* 2007;5:21.

59. Glasgow RE, Nelson CC, Strycker LA, King DK. Using RE-AIM Metrics to Evaluate Diabetes Self-Management Support Interventions. American Journal of Preventive Medicine 2006; 30(1):67-73.

60. Hardeman W, Michie S, Fanshawe T, Prevost AT, McLoughlin K, Kinmonth AL. Fidelity of delivery of a physical activity intervention: Predictors and consequences (2008). *Psychology & Health*, 23, 1, 11-24.

61. Kinmonth AL, Woodcock A, Griffin S, Spiegal N, Campbell MJ. Randomised controlled trial of patient centred care of diabetes in general practice: impact on current wellbeing and future disease risk. *BMJ* 1998;317:1202-8.

62. Montgomery AA, Peters TJ, Little P: Design, analysis and presentation of factorial randomised controlled trials. *BMC Med Res Methodol* 2003;3:26.

63. McAlister FA, Straus SE, Sackett DL, Altman DG: Analysis and reporting of factorial trials: a systematic review. *Jama* 2003;289:2545-2553.

64. White IR & Thompson SG. Adjusting for partially missing baseline measurements in randomized trials. Statistics in Medicine 2005;24:993-1007.

65. Schafer JL. Multiple Imputation: a primer. Statistical Methods in Medical Research 1999;8:3-15.

66. Simmons D, Cheung NW, Lagstrom J, Flack JR, McIntyre HD, Bond D, Johnson E, Wolmarans L, Wein P, Sinha AK for the ADIPS National Diabetes in Pregnancy Audit Project

team. The ADIPS Pilot National Diabetes in Pregnancy Audit Project. Aust NZ J Obs Gynae 2007;47:198-206

67. Simmons D, Coppell K, Drury PL.. The development of a national agreed minimum diabetes dataset for New Zealand. J Qual Clin Pract. 2000;20:44-50.
68. Simmons D, Mesui J. Decisional balance and stage of change in relation to weight loss, exercise and dietary fat reduction among Pacific Islands people. Asia Pacific J Clin Nutr. 1999;8:39-45.
69. Paddison, C.A.M., Alpass, F.M., & C.V. Stephens. Psychological variables account for variation in metabolic control and quality of life among people with type 2 diabetes in New Zealand. *International Journal of Behavioural Medicine* , 2008 (in press)
70. Piatt G, Orchard T, Emerson S, Siminerio L, Simmons D, Korytkowski M, Ahmad U, Songer T, Zgibor J, Brooks M. Translating the Chronic Care Model into the Community: Results from a Randomized Controlled Trial of a Diabetes Education Intervention. Diabetes Care. 2006;29:811-817

Appendix 1 Factors in Bandura's social cognitive theory (24,25)

Environment*:* Factors physically external to the person; provides opportunities and social support

Situation*:* Perception of the environment; correct misperceptions and promote healthful forms

Behavioral capability*:* Knowledge and skill to perform a given behavior; promote mastery learning through skills training

Expectations*:* Anticipatory outcomes of a behavior; model positive outcomes of healthful behavior

Expectancies*:* The values that the person places on a given outcome, incentives; present outcomes of change that have functional meaning

Self-control*:* Personal regulation of goal-directed behavior or performance; provide opportunities for self-monitoring, goal setting, problem solving, and self-reward

Observational learning*:* Behavioral acquisition that occurs by watching the actions and outcomes of others' behavior; include credible role models of the targeted behavior

Reinforcements*:* Responses to a person's behavior that increase or decrease the likelihood of reoccurrence; promote self-initiated rewards and incentives

Self-efficacy*:* The person's confidence in performing a particular behavior; approach behavioral change in small steps to ensure success

Emotional coping responses*:* Strategies or tactics that are used by a person to deal with emotional stimuli; provide training in problem solving and stress management

Reciprocal determinism*:* The dynamic interaction of the person, the behavior, and the environment in which the behavior is performed; consider multiple avenues to behavioral change, including environmental, skill, and personal change.

Appendix 2 Barriers framework (5,6) used in the initial patient survey and how it can be used to provide a framework to generate discussion between peer and person with diabetes in either a group or 1:1 situation.

BARRIER TO CARE	DESCRIPTION (EXAMPLES)	Example of Strategy that peers could use
PSYCHOLOGICAL		
Spiritual health belief	Believe cause/cure should be sought spiritually/within	Where possible gain support from relevant religious authority
Alternative health belief	Prefers uses alternative health models/treatments	Emphasise importance of maintaining treatments advised by health care professionals
Public health belief	Believes the public should bear more financial responsibility for health care	Emphasise importance of self care independent of who pays
Self factors – motivation	Psychological – motivation, attitudes, laziness, denial	Motivational interviewing approaches to key issues
Self factors – self efficacy	No confidence, external locus of control,<br>low self-efficacy	Motivational interviewing approaches to<br>key issues
No symptom cue	No physical symptoms	Emphasise need to prevent future harm-Provide support in continuing self care
Priority setting	Others needs priority over own (e.g. children, elders)	Provide strategies to help prioritise diabetes-reminder where appropriate
Negative perceptions of time	Not enough time (education provided too quickly)	Provide strategies to help prioritise diabetes-reminder where appropriate
Emotional	Fear, shame emotion anxiety, worry – lack of hope	Discuss and support-emphasise that this is normal and how to overcome
Precontemplative	Strictness of regime, giving up things I enjoy	Working through strategies to achieve lifestyle and other goals
EDUCATIONAL		
Low diabetes knowledge	Lacks general/specific diabetes knowledge	Where to obtain information and who to see
Low knowledge of service	Unaware of services available	Explanation of how to navigate through health system and provision of support if need be
INTERNAL PHYSICAL		
Self factors/other health conditions	Diabetes (e.g. amputation) and non-diabetes related (e.g. arthritis)	Support for discussing with health professionals
Physical effects of treatment	Pain of glucose monitoring, Drug side-effects	Support for discussing with health professionals

EXTERNAL PHYSICAL		
Personal finance	Income in relation to costs	Advice in ways to obtain benefits, reduce costs
Service/physical access	Transportation, wheelchair entry	Advice in ways to obtain transport
Unhelpful health professional in past	Past encounter with health professional leading to conflict or without expected communication or clinical expertise	Emphasise importance of self care independent of past problems, support with problem solving re: overcoming any continuing problems
PSYCHO-SOCIAL		
Unsatisfactory/ inappropriate diabetes care or education	Wrong information provided or information provided in inappropriate way	Emphasise importance of self care independent of past problems, support with problem solving re: overcoming any continuing problems
Group pressure	Pressure from others not to adhere to advice	Support to "do the right thing"
Prejudice	Impression of discriminatory practice due to diabetes or for other reasons	Advice on ways to overcome any discrimination
Lack of family support	Family consumes diabetic food, resists change of lifestyle	Advice on strategies to increase family support
Family demands	Pressure to spend time/money on the family rather than their diabetes	Advice on how to reduce family demands
Unsupportive macro-environment	Feeling of lack of support in the community, e.g. access to low fat foods	Advice on how to obtain support
Inappropriate cultural messages	Attitude, ethnicity of workers, appropriateness of communication	Emphasise importance of self care, support with problem solving re: overcoming any continuing problems- could accompany patient

Appendix 2 Programme from Dublin Trial (22)

**MEETING 1**<br>**INTRODUCTION**<br>• Introduction to each other<br>• What is peer support?<br>• Ground rules<br>• Discussion on course content (9 sessions)<br>• Video/DVD 15 mins<br>• Entitlements in diabetes<br>• Identifying a substitute peer supporter<br>• Contact details for the group	**MEETING 2**<br>**HEART AND VASCULAR DISEASE**<br>• Why is it so important?<br>• How you can reduce your risk of heart disease and other vascular complication<br>○ Hypothetical individual and what they would advise them to do<br>Questions relating to heart disease including blood pressure and cholesterol medication and taking tablets
**MEETING 3**<br>**BLOOD SUGAR LEVELS**<br>• Information on hypo/hyperglycaemia<br>• Blood sugar testing<br>Questions on blood sugar levels<br>What to do when you are sick	**MEETING 4**<br>**HEALTHY EATING**<br>Discussion of healthy ‘eating plate’<br>• Laminated picture of the ‘healthy plate’<br>Healthy eating quiz and discussion of answers<br>Questions on healthy eating in diabetes
**MEETING 5**<br>**MEDICATION**<br>• Control of type 2 diabetes<br>○ Diet<br>○ Tablets<br>○ Insulin<br>Questions regarding medication including side effects	**MEETING 6**<br>**EXERCISE**<br>• Importance of exercise<br>• Use of pedometer<br>○ Each person will be given a pedometer<br>Questions about exercise<br>Maybe arrange a walk in locality
**MEETING 7**<br>**FOOT CARE**<br>• Why foot care matters in diabetes<br>• Discussion on how to check feet<br>○ Laminated sheet to cover all aspects of foot care<br>Questions relating to the feet<br>Information on local chiropody services	**MEETING 8**<br>**EYE AND KIDNEY COMPLICATIONS**<br>• What happens to the eyes and kidneys in diabetes<br>• Importance of good blood pressure and blood sugar control in order to prevent complications<br>Questions relating to eye and kidney disease
**MEETING 9**<br>**LIVING WITH DIABETES**<br>This is intended to be a relatively open session in which the group can discuss any remaining concerns and consider whether they would like to continue to meet<br>Importance of follow up data collection!	

Appendix 4 Timeline

Months	Trial Administration	Intervention	Evaluation
1-8	Prepare Participant information leaflets	Develop educational materials including website	Formative evaluation
	Secure ethics approval	Finalise criteria and process for selection of peers	Select and customize measurement instruments
	Recruit Project Manager and lead diabetes specialist nurse for pilot	Finalise curriculum and peer support programme	Develop qualitative evaluation, intervention fidelity evaluation and economic evaluation.
	Recruit and train evaluation staff	Identify areas in Cambridge for 3 month pilots	Identify community networks for recruitment
	Advertise for intervention team	Pilot training one peer group and 1:1 peer support set up in Cambridge	Finalise baseline barriers survey
	Create database		Approach practices to participate in the study
			Commence mail survey
9-12	Liaison with GPs, Community		Complete mail survey
	Recruit intervention team-start month 10	Identify peers	Recruit participants
	Data entry and data checking	Train peers	Undertake baseline participant assessments inc HbA1c
		Roll out educational tools (all groups)-month 11	Obtain health utilization data
		Roll out peer interventions -month 11-12	
13-16		Complete roll out of interventions months 13-14	Collect observational/ qualitative data
		Intervention underway-complete structured programme	
17-18	Data entry and data checking	Intervention underway-complete structured programme	Mid-point questionnaire (mail/telephone/web based)
			HbA1c collection
19-22		Ongoing Peer contact/support programme	Collect observational/ qualitative data
23-26		Complete trial	Final data collection point
			Obtain health utilization data
			Collect qualitative data
27-32	Data entry and data checking	Support peers	Analyse data, write reports
		Translation/Handover	
